# Moderate protein intake percentage in mice for maintaining metabolic health during approach to old age

**DOI:** 10.1007/s11357-023-00797-3

**Published:** 2023-04-28

**Authors:** Yoshitaka Kondo, Hitoshi Aoki, Masato Masuda, Hiroki Nishi, Yoshihiro Noda, Fumihiko Hakuno, Shin-Ichiro Takahashi, Takuya Chiba, Akihito Ishigami

**Affiliations:** 1 Molecular Regulation of Aging, Tokyo Metropolitan Institute of Gerontology, 35-2 Sakae-Cho, Itabashi-Ku, Tokyo, 173-0015 Japan; 2 Biomedical Gerontology Laboratory, Faculty of Human Sciences, Waseda University, Saitama, 359-1192 Japan; 3 Research and Development Division, Nichirei Foods Inc, Chiba, 261-0002 Japan; 4 Department of Animal Sciences and Applied Biological Chemistry, Graduate School of Agricultural and Life Sciences, University of Tokyo, Tokyo, 113-8657 Japan; 5 Department of Animal Facility, Tokyo Metropolitan Institute of Gerontology, Tokyo, 173-0015 Japan

**Keywords:** Aging, Amino acid, Fatty liver, Metabolic health, Macronutrients, Protein

## Abstract

Nutritional requirements for maintaining metabolic health may vary with each life stage, such as young, middle, and old age. To investigate the appropriate ratio of nutrients, particularly proteins, for maintaining metabolic health while approaching old age, young (6-month-old) and middle-aged (16-month-old) mice were fed isocaloric diets with varying protein percentages (5%, 15%, 25%, 35%, and 45% by calorie ratio) for two months. The low-protein diet developed mild fatty liver, with middle-aged mice showing more lipids than young mice, whereas the moderate-protein diet suppressed lipid contents and lowered the levels of blood glucose and lipids. Self-organizing map (SOM) analysis revealed that plasma amino acid profiles differed depending on age and difference in protein diet and were associated with hepatic triglyceride and cholesterol levels. Results indicate that the moderate protein intake percentages (25% and 35%) are required for maintaining metabolic health in middle-aged mice, which is similar to that in young mice.

**Supplementary information:**

The online version contains supplementary material available at 10.1007/s11357-023-00797-3.

## Introduction

In recent years, the extending of health span has been accepted as a goal of research on aging in many countries, where the number of elderly people is increasing [1, 2]. Lifespan is defined as “how long one lives” and healthspan as “the healthy periods, without diseases, of one’s life”. Therefore, various nutritional and pharmacological methodologies have been proposed to delay the aging process and prevent age-related diseases such as cancer, dementia, metabolic syndrome, diabetes mellitus, and cardiovascular diseases [3]. In particular, the prevention and improvement of emerging diseases such as sarcopenia [4], frailty [5–7], and non-alcoholic fatty liver disease (NAFLD) [8], are important issues to be addressed in this context.

Nutritional interventions, such as calorie and protein restrictions and intermittent fasting, are known to increase the healthspan and lifespan of primates and rodents [9–11]. However, the feasibility of these interventions in humans is problematic. Recently, researchers have found a plausible way to vary the ratio of dietary macronutrients, such as proteins, carbohydrates, and fat in rodents [11–15]. Solon-Biet et al*.* have reported that the ratio of dietary macronutrients (protein, carbohydrate, and fat), and not caloric intake, dictate cardiometabolic health and aging, and also that low-protein and high-carbohydrate diets are associated with the longest lifespan in lifelong ad libitum-fed mice [16]. In contrast, adequate dietary protein intake is recommended to prevent frailty and sarcopenia in humans [17, 18]. Therefore, the optimal balance of macronutrients for ideal health outcomes may vary across different life stages. Senior et al*.* showed the possibility of minimizing age-specific mortality throughout life by changing the ratio of dietary protein to carbohydrate during approach to old age in mice [12].

In addition, more recent studies on dietary macronutrients and aging have focused on nutritional memory, sex, and the genetic background of mice [11, 19, 20]. Hahn et al*.* reported that late-life dietary restriction, the switch from dietary restriction to ad libitum at 24 months of age, increased mortality in female mice, and the switch from ad libitum to dietary restriction slightly increased survival, which considers nutritional memory [20]. Moreover, Green et al*.* showed that sex and genetic background are key factors in the response to protein restriction using a multi-omics approach [19].

However, the amount of protein that should be consumed to maintain metabolic health while approaching old age is still unclear. To determine the same, we fed young and middle-aged male mice diets containing different protein contents [5% (P5), 15% (P15), 25% (P25), 35% (P35), and 45% (P45) by calorie ratio] and investigated the weight dynamics of individual skeletal muscles, such as the gastrocnemius (Gas), tibialis anterior (TA), plantaris (Pla), extensor digitorum longus (EDL), soleus (Sol), lipid profile in liver and plasma, and amino acid composition in plasma. Furthermore, in this study, a self-organizing map (SOM) [21] revealed that plasma amino acid concentrations depended on age and different protein diets, and were associated with hepatic lipid content in young and middle-aged male mice which were fed diets containing different protein contents.

## Materials and methods

### Animals and diets

Animal experiments were conducted in accordance with animal care and protocols approved by the Institutional Animal Care and Use Committee of the Tokyo Metropolitan Institute of Gerontology (TMIG, Tokyo, Japan) (Permit Number:17059) and the Guidelines for the Care and Use of Laboratory Animals of TMIG. Male C57BL/6NCr mice at 6- and 16-months of age were fed a Charles River Formula-1 (CRF-1) diet (Supplemental Table 1) (Oriental Yeast, Tokyo, Japan) and obtained from the animal facility of TMIG. Mice were divided into five groups: P5, P15, P25, P35, and P45. Mice in each group were fed modified AIN-93 M mature rodent diets with 5, 15, 25, 35, or 45 kcal% protein; 70, 60, 50, 40, or 30 kcal% carbohydrate; and 25 kcal% fat (Research Diets, Inc., NJ, USA), as shown in Table 1. Body weight, food consumption, and water consumption were measured every 2 weeks. Throughout the experiments, the animals were maintained under a 12-h light/dark cycle in a controlled environment. The number of animals used was kept at the minimum necessary for meaningful interpretation of the data. Animal discomfort was kept to a minimum.

**Table 1:** Diet composition of animal experiment

Group	P5		P15		P25		P35		P45	
	g%	kcal%	g%	kcal%	g%	kcal%	g%	kcal%	g%	kcal%
Protein	5	5.0	16	15.0	26	25.0	37	35.0	47	45.0
Carbohydrate	73	70.0	63	60.0	53	50.0	42	40.0	32	30.0
Fat	12	25.0	12	25.0	12	25.0	12	25.0	12	25.0
Total		100.0		100.0		100.0		100.0		100.0
kcal/g	4.2		4.2		4.2		4.2		4.2	
Ingredient	g	kcal	g	kcal	g	kcal	g	kcal	g	kcal
Casein	47.6	190	142.5	570	237.5	950	332.5	1330	427.6	1710
L-Cystine	0.6	2	1.8	7	3.1	12	4.3	17	5.5	22
Corn starch	438.5	1754	342.5	1370	246.2	985	150.0	600	53.75	215
Maltodextrin 10	125	500	125	500	125	500	125	500	125	500
Sucrose	100	400	100	400	100	400	100	400	100	400
Cellulose, BW200	50	0	50	0	50	0	50	0	50	0
Soybean oil	107	963	107	963	107	963	107	963	107	963
t-Butylhydroquinone	0.0214	0	0.0214	0	0.0214	0	0.0214	0	0.0214	0
Mineral mix S10022M	35	0	35	0	35	0	35	0	35	0
Potassium phosphate	3.25	0	0	0	0	0	0	0	0	0
Vitamin mix V10037	10	40	10	40	10	40	10	40	10	40
Choline bitartrate	2.5	0	2.5	0	2.5	0	2.5	0	2.5	0
FD&C yellow #5	0.025	0	0.05	0	0	0	0	0	0	0
FD&C red #40	0	0	0	0	0.05	0	0	0	0.025	0
FD&C blue #1	0.025	0	0	0	0	0	0.05	0	0.025	0
Total	919.5	3850	916.4	3850	916.4	3850	916.4	3850	916.4	3850

### Dietary amino acids quantification

Dietary amino acids were measured using an amino acid automatic analysis method and high-performance liquid chromatography (HPLC) in the laboratory of Japan Food Research Laboratories (Tokyo, Japan).

### Tissue harvest

All mice were kept on fast for three hours before being euthanized. Blood was collected from the inferior vena cava, anticoagulated with ethylenediaminetetraacetic acid, and centrifuged at 880 × *g* for 15 min at 4 °C. The supernatant was collected as plasma samples. Blood was collected from the tail vein for glucose assay. Mice were perfused with ice-cold phosphate-buffered saline through the left ventricle to wash out the blood, and the liver, kidneys, and skeletal muscles, including the gastrocnemius (Gas), tibialis anterior (TA), plantaris (Pla), extensor digitorum longus (EDL), and soleus (Sol) were removed. The livers were frozen in liquid nitrogen for biochemical analysis. The liver and kidneys were fixed in 10% neutral buffered formalin (Mildform® 10N, FUJIFILM Wako Pure Chemical Corp., Osaka, Japan) for histological analysis. The formalin-fixed samples were stored at 4 °C, and the other samples were stored at -80 °C until use.

### Histology

To evaluate histological changes in the livers and kidneys of mice fed diets containing different protein contents (5–45%), fixed liver sections were subjected to HE staining. Kidney sections were subjected to PAS staining to evaluate renal degeneration.

### Hepatic lipid quantification

Triglyceride, total cholesterol, free cholesterol, and cholesterol ester levels in total lipid extracts from the liver were quantified using the Folch method [22] in the laboratory of Skylight Biotech, Inc. (Akita, Japan).

### Plasma biochemistry

The levels of triglycerides, total cholesterol, high-density lipoprotein cholesterol, low-density lipoprotein cholesterol, free fatty acids, total ketone bodies, blood urea nitrogen, aspartate aminotransferase, alanine aminotransferase, total protein, albumin, albumin/globulin, and creatinine in the plasma were analyzed using biochemical analysis of blood services (Oriental Yeast Co., Ltd., Tokyo, Japan). Blood glucose levels were measured using a blood glucose meter (Glutest Every; Sanwa Kagaku Kenkyusho Co. Ltd., Nagoya, Japan).

### Plasma amino acids

Plasma amino acids were measured using the method described by Shimbo et al. [23] through pre-column derivatization high-performance liquid chromatography/electrospray mass spectrometry (HPLC/ESI‐MS) in the laboratory of SRL Inc. (Tokyo, Japan).

### Self-organizing map (SOM) cluster analysis of plasma amino acid profiles

SOM analysis was performed as previously described [21]. Briefly, an initial map was prepared consisting of 40 × 40 units, each carrying a random vector. The Euclidean distances between an input vector containing the plasma amino acid concentrations of a mouse for the training data and all units on a map were calculated. One unit with minimal distance (winner unit) was chosen, and then the vectors on it and its neighborhood units were modified to make them closer to the input vector, which was applied to all the input data. However, only amino acids were used to select the winner units. For the learning parameters, three patterns of 0.2 → 0.01, 0.5 → 0.1, and 0.7 → 0.2 were used for the learning coefficients, and three patterns of 5 → 1, 10 → 5, and 20 → 10 were used for the neighborhood radius. Maps were connected at the top and bottom and left and right (periodic boundary condition). These operations were repeated 10,000 times to obtain a final map.

### Quantification and statistical analysis

Data are presented as mean ± SEM. All statistical analyses were performed using GraphPad Prism 7.03 (GraphPad Software, CA, USA). Statistical analyses for age (young and middle-aged) were performed using the Welch’s t-test. Linear regression analyses were used to analyze food, protein, fat, and carbohydrate intake in young and middle-aged mice. Statistical analyses for the overall effect of diet, age, and the interaction were performed using a two-way ANOVA, followed by a Sidak post-test to examine the effect of age or Tukey post-test to examine the effects of diet. The sample sizes and other statistical parameters are shown in the figures. Statistical significance was set at *p* < 0.05 (^*^ *p* < 0.05, ^**^ *p* < 0.01, ^***^ *p* < 0.001).

## Results

### Survival curve of mice fed CRF-1 diet for their entire lifetime and their metabolic state

**Fig. 1:**
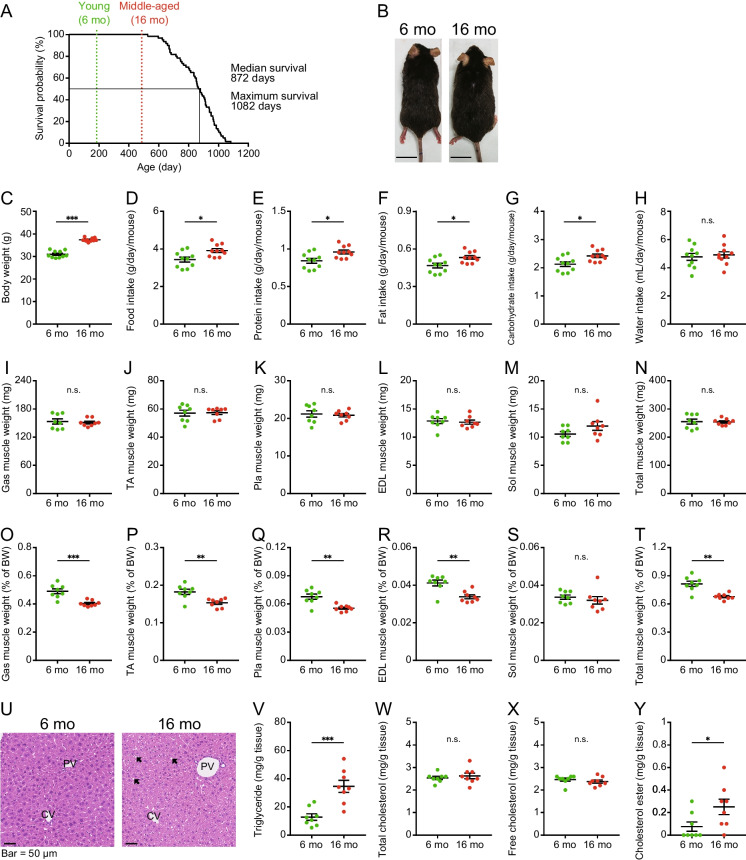
Survival curve of C57BL/6NCr male mice and physiological parameters of young and middle-aged mice. (**A**) Kaplan–Meier survival curves of the C57BL/6NCr male mice (n = 60) fed a CRF-1 diet (Supplemental Table 1). (**B**) Representative appearance of young (6 mo: 6 months of age) and middle-aged mice (16 mo: 16 months of age). (**C**) Bodyweight, (**D**) Food intake, (**E**) protein intake (**F**) fat intake, (**G**) carbohydrate intake, and (**H**) water intake, in young and middle-aged mice of cages (n = 10 per group). Weights of the (**I**) gastrocnemius (Gas), (**J**) tibialis anterior (TA), (**K**) plantaris (Pla), (**L**) extensor digitorum longus (EDL), and (**M**) soleus (Sol) muscles, and (**N**) total weight of five muscles of young and middle-aged mice (n = 8 per group). Weights normalized by bodyweight of (**O**) Gas muscle, (**P**) TA muscle, (**Q**) Pla muscle, and (**R**) EDL muscle, (**S**) Sol muscle, and (**T**) total weight of five muscles in young and middle-aged mice (n = 8 per group). (**U**) Representative images of hematoxylin–eosin (HE) staining in the liver section from young and middle-aged mice. PV, portal vein; CV, central vein. Where, black arrows indicate lipid droplets. (**V**) Content of hepatic triglycerides, (**W**) total cholesterols, (**X**) free cholesterols, and (**Y**) cholesterol esters in young and middle-aged mice (n = 8 per group). Data are presented as mean ± SEM and analyzed using the Welch’s t-test (C-T, V–Y). n.s., not significant. ^*^ *p* < 0.05, ^**^ *p* < 0.01, ^***^ *p* < 0.001

Kaplan–Meier survival curves of C57BL/6NCr male mice that were fed a CRF-1 diet (Supplemental Table 1) for their entire lifetime are shown in Fig. 1A. The median and maximum survival times were 872 days (29 months) and 1,082 days (36 months), respectively. The first mice that died were 526 days old (17.5 months of age). The appearance of young (6 month-old) and middle-aged (16 month-old) mice used in this study is shown in Fig. 1B.

The body weight of middle-aged mice was significantly higher than that of young mice (Fig. 1C). Food intake was significantly higher in middle-aged mice than in young mice (Fig. 1D). We calculated the actual protein, fat, and carbohydrate intakes from their diet. The protein, fat, and carbohydrate intakes of the middle-aged mice were significantly higher than in young mice (Fig. 1E-G). There were no significant differences in water intake between young and middle-aged mice (Fig. 1H).

We examined the effect of aging on the weight of skeletal muscles such as the Gas, TA, Pla, EDL, and Sol muscles in the legs. There was no significant difference in muscle weight between young and middle-aged mice (Fig. 1I-N). However, there were significant differences in muscle weights normalized by body weight between young and middle-aged mice, except in Sol muscles (Fig. 1O-T).

Next, we examined the effects of aging on lipid accumulation in the livers of young and middle-aged mice. Hematoxylin–eosin (HE) staining of liver sections showed fatty vacuolations in the perilobular hepatocytes of middle-aged mice (Fig. 1U). To characterize the hepatic lipid composition, we quantified triglycerides, total cholesterol, free cholesterol, and cholesterol esters in the livers. Compared to young mice, the hepatic triglyceride and cholesterol ester contents were significantly higher in middle-aged mice, although there were no significant differences in the hepatic total cholesterol and free cholesterol contents between young and middle-aged mice (Fig. 1V-Y).

We also examined the effects of aging on blood biochemical parameters and plasma amino acid concentrations in young and middle-aged mice (Supplemental Fig. 1). Plasma HDL-cholesterol levels were significantly higher in middle-aged mice than in young mice, whereas plasma aspartate aminotransferase (AST) and alanine aminotransferase (ALT) levels were significantly lower in middle-aged mice than in young mice (Supplemental Fig. 1G-I). Plasma valine levels in middle-aged mice were significantly lower than those in young mice, while plasma cystine and alanine levels in middle-aged mice were significantly higher than those in young mice (Supplemental Fig. 1P, X, and AC).

To evaluate the effect of aging on the kidneys, we examined kidney sections in young and middle-aged mice using periodic acid-Schiff (PAS) staining. No morphological abnormalities, such as shrinking glomerular and tubular lesions, were observed in the glomeruli and renal tubules (Supplemental Fig. 1AP).

### Different protein diets, with equal calories, affect body weight and food intake in mice

**Fig. 2:**
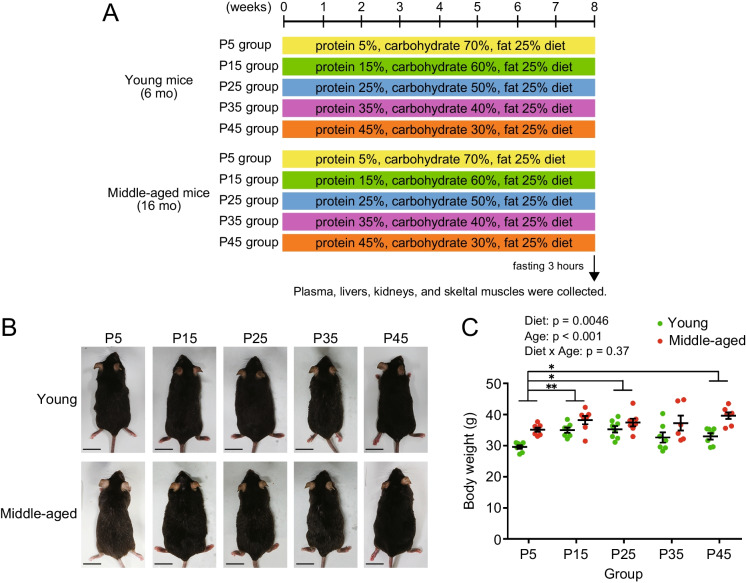
Animal experimental design and bodyweight of mice. (**A**) Six- (young) and 16-month (middle-aged) old mice were divided into five groups: P5, P15, P25, P35, and P45, which consumed diets with the same lipid content (25 kcal% fat) but different carbohydrate (70, 60, 50, 40, or 30 kcal% carbohydrate, respectively) and protein contents (5, 15, 25, 35, or 45 kcal% protein, respectively) as shown in Table 1. Also refer to Supplemental Fig. 2. (**B**) Representative appearance of mice after 8 weeks of feeding. (**C**) Body weight of young and middle-aged mice (*n* = 6–8 per group) at 8 weeks of feeding. Data are presented as mean ± SEM and analyzed using two-way ANOVA followed by Tukey post-test to examine the effects of diet (**C**). ^*^ *p* < 0.05, ^**^ *p* < 0.01

**Fig. 3:**
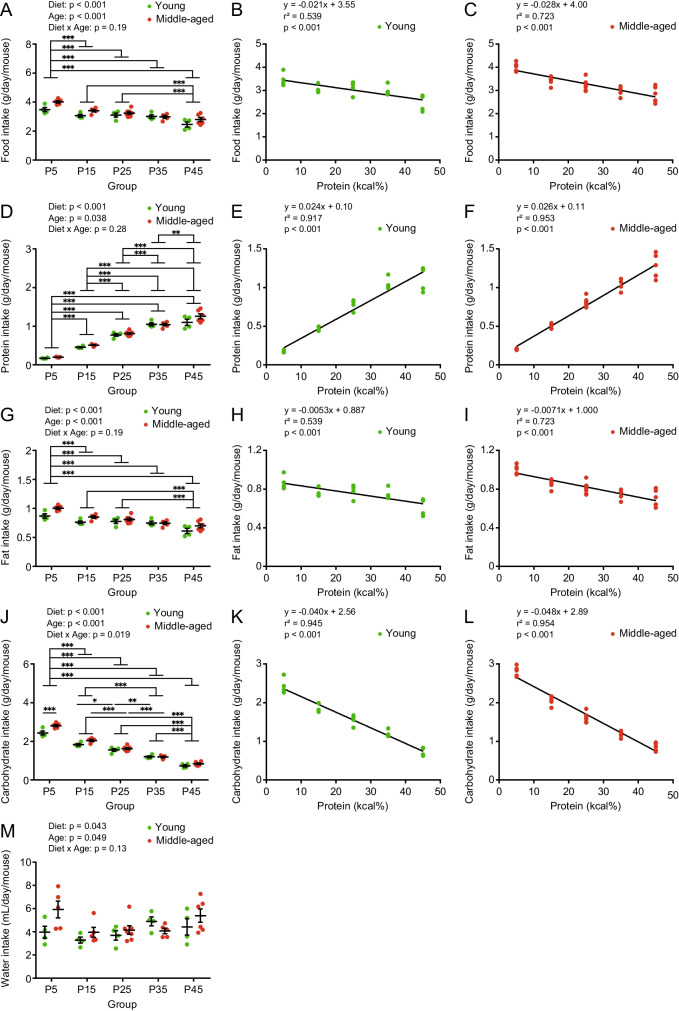
Food intake of young and middle-aged mice fed with different protein diets. (**A**, **B**, **C**) Food intake, (**D**, **E**, **F**) protein intake (**G**, **H**, **I**) fat intake, (**J**, **K**, **L**) carbohydrate intake, and (**M**) water intake, in young and middle-aged mice of cages (n = 4–7 per group) after 8 weeks of feeding. (**B**, **C**) Regression line of food intake, (**E**, **F**) protein intake, (**H**, **I**) fat intake, (**K**, **L**) carbohydrate intake in young and middle-aged mice of cages after 8 weeks of feeding. Data are presented as mean ± SEM and analyzed using two-way ANOVA followed by a Sidak post-test to examine the effect of age or Tukey post-test to examine the effects of diet (**A**, **D**, **G**, **J**, **M**). Linear regression analyses were performed to examine the association between dietary protein percentage and food (**B**, **C**), protein (**E**, **F**), fat (**H**, **I**), and carbohydrate (**K**, **L**) intake. ^*^ *p* < 0.05, ^**^ *p* < 0.01, ^***^ *p* < 0.001

To investigate the ideal ratio of protein intake for maintaining metabolic health during approach to old age, we took 6-months old (young) and 16-months old (middle-aged) male mice and divided them into five groups: P5, P15, P25, P35, and P45 (Fig. 2A). The mice in these groups were fed diets that consisted of equal calories but different nutrient compositions: 5, 15, 25, 35, or 45 kcal% protein; 70, 60, 50, 40, or 30 kcal% carbohydrate; and 25 kcal% fat (Table 1). Before starting the animal experiments, we validated the dietary amino acid content and confirmed that its proportion was derived from casein (Supplemental Fig. 2). The appearance of mice in each group after 8 weeks of the indicated dietary regimens is shown in Fig. 2B. Overall, the body weight of middle-aged mice was higher than that of young mice (Fig. 2C). The body weights of the P5 group were significantly lower than those of the P15, P25, and P45 groups (Fig. 2C). The mice ate progressively more food as their dietary protein content declined, with middle-aged mice eating more than young mice (Fig. 3A). Moreover, linear regression analysis showed that food intake was dependent on dietary protein content in both young and middle-aged mice (Fig. 3B and C). We then calculated the actual protein, fat, and carbohydrate intake from the food for each group. Overall, protein intake was highly dependent on dietary protein content and was higher in middle-aged mice than in young mice (Fig. 3D-F). Fat intake was calculated as per food intake (Fig. 3G-I). Although carbohydrate intake was also parallel to food intake and highly dependent on the dietary protein content, the middle-aged P5 showed a significantly higher intake than young P5 mice (Fig. 3J-L). Diet and age had a significant effect on water intake (Fig. 3M).

### Overall skeletal muscles weights are unaffected by intake of different protein diets

**Fig. 4:**
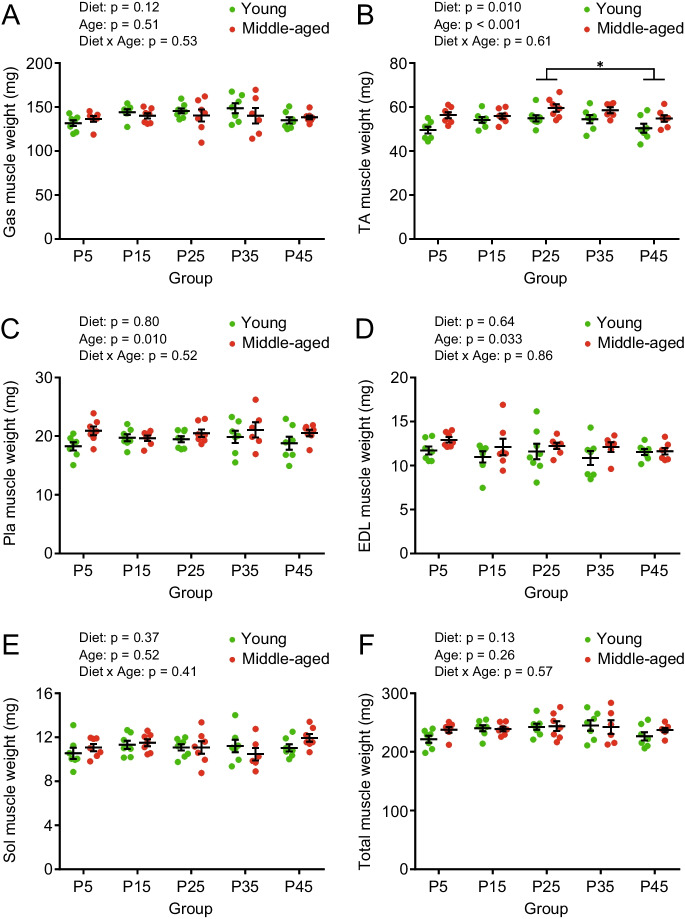
Skeletal muscle weights of young and middle-aged mice fed with different protein diets. Weights of (**A**) gastrocnemius (Gas), (**B**) tibialis anterior (TA), (**C**) plantaris (Pla), (**D**) extensor digitorum longus (EDL), and (**E**) soleus (Sol) muscles and (**F**) total weight of five muscles in young and middle-aged mice (n = 6–8 per group) after 8 weeks of feeding. Data are presented as mean ± SEM and analyzed using two-way ANOVA followed by Tukey post-test to examine the effects of diet (A-F). ^*^ *p* < 0.05. The ratio of skeletal muscle weight to body weight in Supplemental Fig. 3

High protein intake from dietary supplements, meals, or both has been recommended for the prevention and intervention of sarcopenia and frailty [24]. Therefore, we examined the effect of different protein diets on the weight of skeletal muscles such as the Gas, TA, Pla, EDL, and Sol muscles in the legs. Overall, the muscle weights of TA, Pla, and EDL were significantly higher in middle-aged mice than in young mice, and there was a significant diet effect on only TA muscle weight (Fig. 4). In contrast, the muscle weights of Gas, Sol, and total normalized by body weight were significantly lower in middle-aged mice than in young mice, and there were significant dietary effects on TA, EDL, and total muscle weight (Supplemental Fig. 3).

### Moderate protein diet suppressed triglyceride content in the liver of young and middle-aged mice

**Fig. 5:**
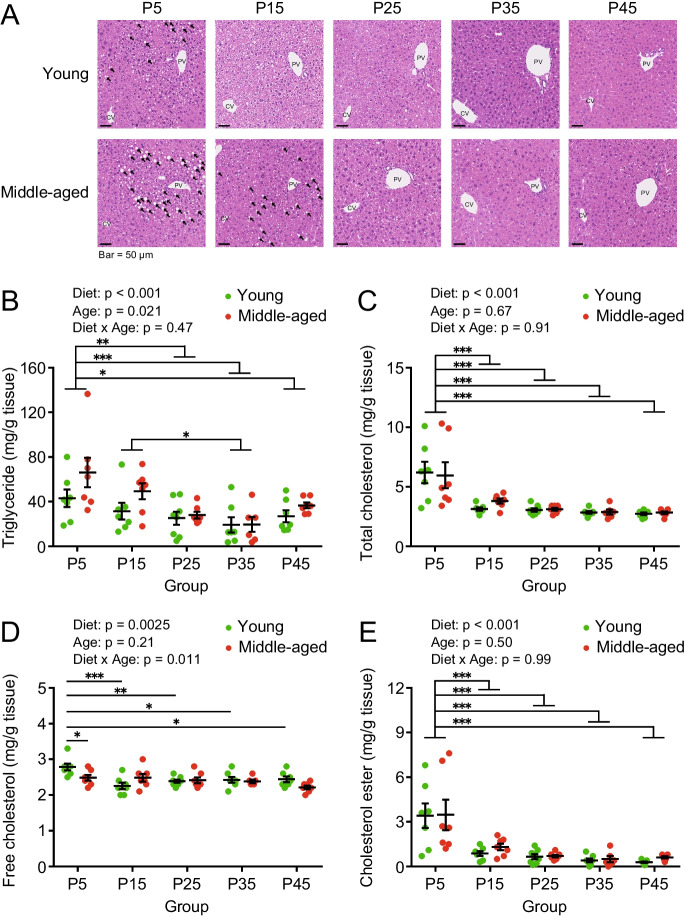
Hepatic lipid contents in young and middle-aged mice fed with different protein diets. (**A**) Representative images of hematoxylin–eosin (HE) staining in the liver section from young and middle-aged mice (*n* = 6–8 per group) fed with diets of different protein contents at 8 weeks of feeding. PV, portal vein; CV, central vein. (**B**) Content of hepatic triglycerides, (**C**) total cholesterols, (**D**) free cholesterols, and (**E**) cholesterol esters in young and middle-aged mice (*n* = 6–8 per group) after 8 weeks of feeding. Data are presented as mean ± SEM and analyzed using two-way ANOVA followed by a Sidak post-test to examine the effect of age or Tukey post-test to examine the effects of diet (B-E). ^*^ *p* < 0.05, ^**^ *p* < 0.01, ^***^ *p* < 0.001

We previously reported that a low-protein diet induced fatty liver and high levels of triglycerides in growing rats [25]. Therefore, we examined the effects of different protein diets and ages on lipid accumulation in the livers of young and middle-aged mice. HE staining of the liver sections showed many fatty vacuolations in the perilobular hepatocytes of the young P5, middle-aged P5, and P15 groups (Fig. 5A). To characterize the hepatic lipid composition, we quantified triglycerides, total cholesterol, free cholesterol, and cholesterol esters in the livers of mice. Overall, the hepatic triglyceride content was significantly higher in middle-aged mice than in young mice (Fig. 5B). Hepatic triglyceride content was significantly higher in the P5 group than in the P25, P35, and P45 groups (Fig. 5B). Hepatic triglyceride levels in the P35 group were significantly lower than those in the P15 group (Fig. 5B). The hepatic contents of total cholesterol and its component cholesterol esters were significantly higher in the P5 group than in the other diet groups (Fig. 5C and E), but there was no significant effect with respect to age. The young P5 group showed significantly higher values of hepatic free cholesterol than the other young and middle-aged P5 groups (Fig. 5D). Compared to young mice, hepatic lipid accumulation in middle-aged mice was found to be relatively susceptible to low-protein diets. Furthermore, the moderate protein diet reduced hepatic triglyceride accumulation in young and middle-aged mice.

### Moderate protein diet as a well-balanced intervention for plasma metabolic parameters

**Fig. 6:**
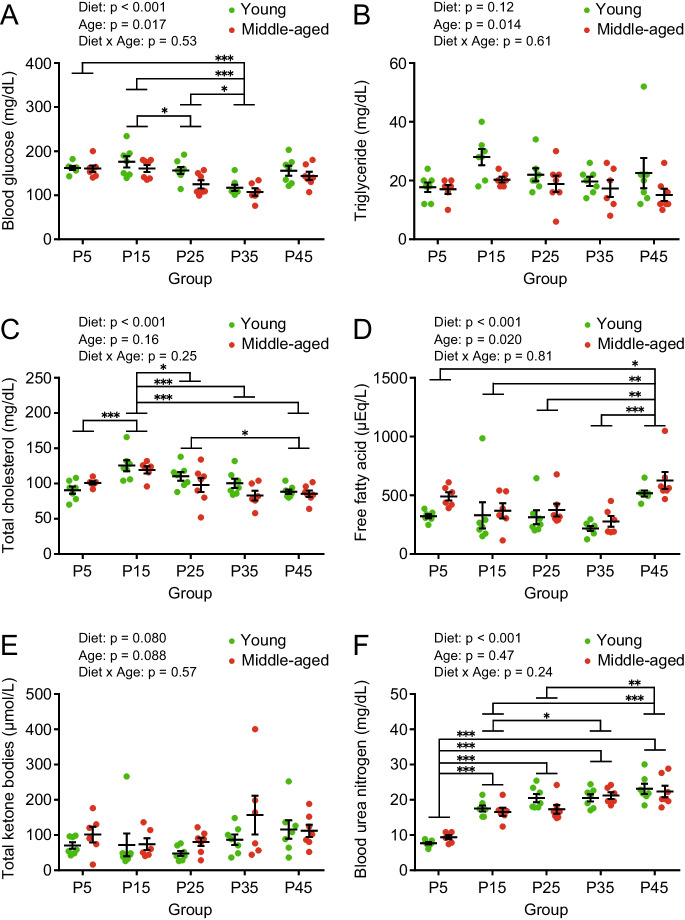
Blood biochemical parameters in young and middle-aged mice fed with different protein diets. The concentrations of (**A**) blood glucose, (**B**) plasma triglycerides, (**C**) total cholesterol, (**D**) free fatty acids, (**E**) total ketone bodies, and (**F**) blood urea nitrogen in young and middle-aged mice (*n* = 6–8 per group) after 8 weeks of feeding. Data are presented as mean ± SEM and analyzed using two-way ANOVA followed by Tukey post-test to examine the effects of diet (A-F). ^*^ *p* < 0.05, ^**^ *p* < 0.01, ^***^ *p* < 0.001

Blood biochemical parameters, such as glucose, triglyceride, and cholesterol levels, are crucial for monitoring the metabolic state and preventing metabolic diseases in clinical and animal studies. Overall, the blood glucose levels were significantly lower in middle-aged mice than in young mice (Fig. 6A). In the P25 group, blood glucose levels were significantly lower than those in the P15 group (Fig. 6A). Blood glucose levels in the P35 group were lower than those in the P5, P15, and P25 groups (Fig. 6A). Plasma triglyceride levels were lower in middle-aged mice than in young mice; however, there was no significant effect based on diet among the groups (Fig. 6B). Although plasma total, LDL, and HDL cholesterol levels did not show a significant effect on age, diet had a significant effect on these parameters (Fig. 6C, Supplemental Fig. 4A, and B). The plasma levels of total and HDL cholesterol in the P5, P25, P35, and P45 groups were significantly lower than those in the P15 group (Fig. 6C and Supplemental Fig. 4B). The overall plasma levels of free fatty acids were higher in middle-aged mice than in young mice, and those in the P45 group were the highest among all the dietary groups (Fig. 6D). There were no significant effects of age or diet on the plasma levels of total ketone bodies (Fig. 6E). Plasma AST and ALT levels, which are markers of hepatocellular injury, were within the normal range (Supplemental Fig. 4C and D). Overall, middle-aged mice showed significantly higher plasma total protein levels and lower albumin/globulin levels than young mice (Supplemental Fig. 4E and G). The P5 and P45 groups had lower plasma total protein and higher albumin/globulin levels than the P15, P25, and P35 groups, respectively (Supplemental Fig. 4E and G). There were no significant effects of age or diet on plasma albumin levels (Supplemental Fig. 4F).

A high-protein diet can be a burden to the kidneys; therefore, it is not recommended for patients with renal impairment. To evaluate the effect of high protein intake on the kidneys, we examined kidney sections from middle-aged mice using PAS staining. No morphological abnormalities, such as shrinking glomerular and tubular lesions, were observed in the glomeruli and renal tubules (Supplemental Fig. 5). Plasma creatinine levels reflect the balance between creatinine production from muscle cells and excretion from the kidneys. Some middle-aged mice showed higher creatinine levels in the P5, P15, P25, and P35 groups; however, creatinine levels in the P45 group were lower than those in the other diet groups (Supplemental Fig. 4H). These results suggest that high protein intake does not cause renal impairment. Blood urea nitrogen levels, which indicate the urea balance between its production from amino acid catabolism and excretion from the kidney, were significantly lower in the P5 group than in the other diet groups, whereas the values in the P45 group were significantly higher than those in the P15 and P25 groups (Fig. 6F). These data suggest that amino acid catabolism may be suppressed in P5 and enhanced in P45. The moderate protein diet is a well-balanced intervention for metabolic health in young and middle-aged mice.

### Different protein diets affect plasma amino acid profiles in young and middle-aged mice

**Fig. 7:**
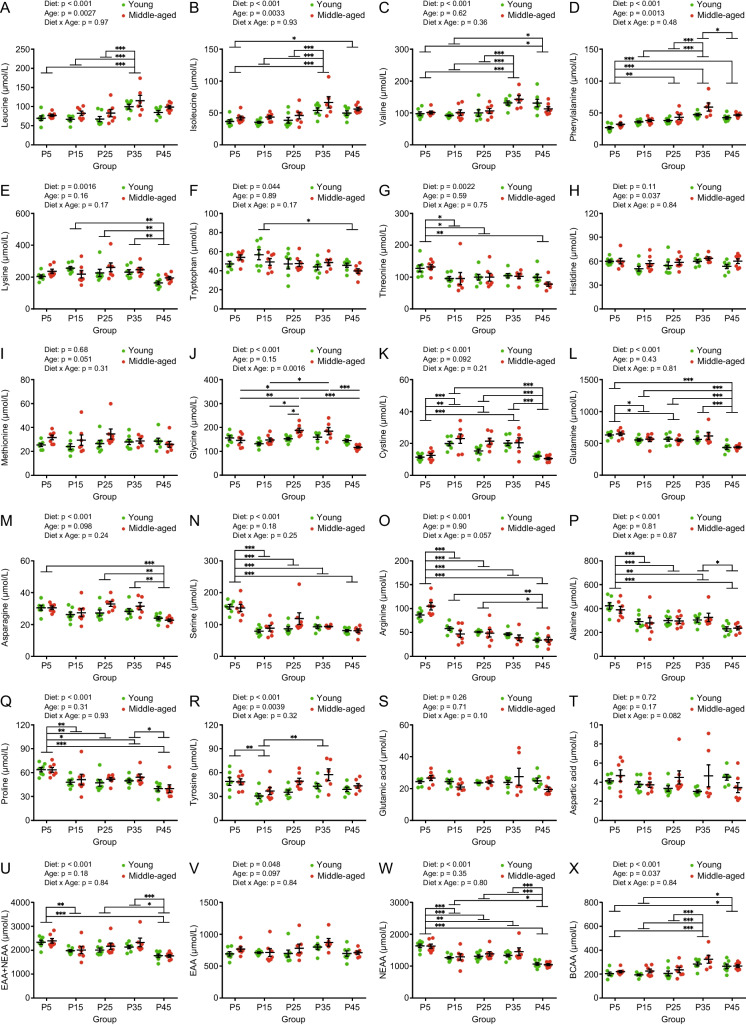
Plasma amino acid concentrations in young and middle-aged mice fed with different protein diets. Plasma concentrations of (**A**) leucine, (**B**) isoleucine, (**C**) valine, (**D**) phenylalanine, (**E**) lysine, (**F**) tryptophan, (**G**) threonine, (**H**) histidine, (**I**) methionine, (**J**) glycine, (**K**) cystine, (**L**) glutamine, (**M**) asparagine, (**N**) serine, (**O**) arginine, (**P**) alanine, (**Q**) proline, (**R**) tyrosine, (**S**) glutamic acid, (**T**) aspartic acid, (**U**) essential amino acid (EAA), non-essential amino acid (NEAA), (**V**) EAA, (**W**) NEAA, and (**X**) branched-chain amino acid (BCAA) in young and middle-aged mice (*n* = 6–7 per group) after 8 weeks of feeding. Data are presented as mean ± SEM and analyzed using two-way ANOVA followed by a Sidak post-test to examine the effect of age or Tukey post-test to examine the effects of diet (**A**–**X**). ^*^ *p* < 0.05, ^**^ *p* < 0.01, ^***^ *p* < 0.001

We examined the effects of different protein diets on the plasma amino acid concentrations in young and middle-aged mice (Fig. 7 and Supplemental Fig. 6). Among the 20 constituent amino acids, nine essential amino acids (EAAs)–leucine, isoleucine, valine, phenylalanine, lysine, tryptophan, threonine, histidine, and methionine–cannot be synthesized in mice and humans; therefore, they must be obtained from the diet. The plasma concentrations of branched-chain amino acids (BCAA), leucine, isoleucine, and valine were significantly higher in the P35 and P45 groups than in the other groups, with an overall higher value in middle-aged mice (Fig. 7X). The plasma concentration of EAAs showed a pattern similar to that of BCAA, but only the diet effect was observed (Fig. 7V). Eleven types of non-essential amino acids (NEAAs), including glycine, cysteine (measured as cystine), glutamine, asparagine, serine, arginine, alanine, proline, tyrosine, glutamic acid, and aspartic acid, are synthesized in the body. Overall, the plasma concentrations of NEAAs were significantly higher in the P5 group and lower in the P45 group than in the P15, P25, and P35 groups; however, there was no effect of age (Fig. 7W). The total plasma concentrations of EAAs and NEAAs showed patterns similar to those of NEAAs (Fig. 7U).

The plasma concentrations of leucine, isoleucine, phenylalanine, histidine, and tyrosine were significantly higher in middle-aged mice than in young mice (Fig. 7A, B, D, H and R). There were significant effects of diet on leucine, isoleucine, valine, phenylalanine, lysine, tryptophan, threonine, cystine, glutamine, asparagine, serine, arginine, alanine, proline, and tyrosine (Fig. 7A-G and K-R). Glycine levels showed an interaction between diet and age (Fig. 7J). There were no significant differences in the methionine, glutamic acid, and aspartic acid levels (Fig. 7I, S, and T). Since plasma concentrations of each amino acid were distinctively affected by different protein diets and ages, it was necessary to understand the plasma amino acid profiles in individual mice.

### Plasma amino acid profiles depended on age and diet

**Fig. 8:**
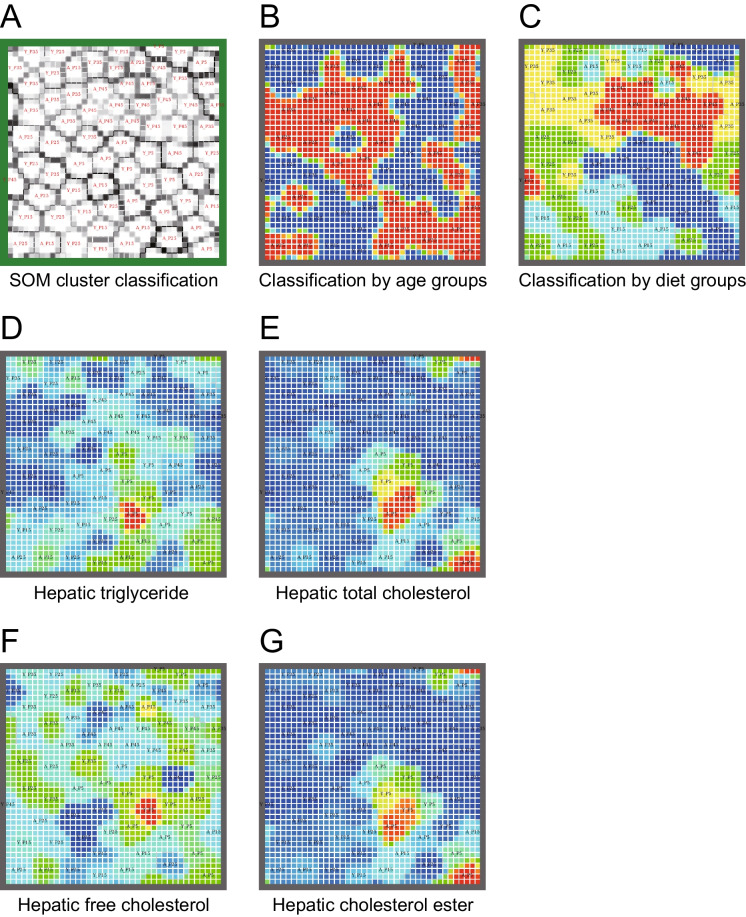
Self-organizing map (SOM) of plasma amino acid profiles classified by age and different protein diets and associated with hepatic lipid contents in young and middle-aged mice. (**A**) SOM cluster classification. The map was obtained using plasma amino acid concentrations of each mouse as input vectors. Similar vectors were placed near each other, forming clusters. Color and line represent the distance between neighborhood vectors (white, close: black, far). (**B**) Classification of groups by age (blue, young; red, middle-aged) and (**C**) diets (blue, P5; light blue, P15; green, P25: yellow, P35; red, P45) in the map obtained in (**A**). Heat map of (**D**) hepatic triglycerides, (**E**) total cholesterols, (**F**) free cholesterols, and (**G**) cholesterol esters contents overlaid on the map obtained in (**A**). Colors represents measured normalized values of triglycerides or cholesterols contents (red, high = 1; blue, low = 0). Data sets of plasma amino acid concentrations and hepatic lipid contents were obtained from 69 young and middle-aged mice (n = 6–7 per group) after 8 weeks of feeding

In SOM cluster analysis, the map was obtained using the plasma amino acid concentrations of each mouse as input vectors (Fig. 8A). The SOM analysis map uses periodic boundary conditions. Similar vectors are placed near each other, forming clusters. To examine the relationship between age and diet, heat maps of age and diet were overlaid on the SOM (Fig. 8B and C). As shown in Fig. 8B, each mouse was classified by plasma amino acid profile and age, suggesting that the plasma amino acid profiles changed in an age-dependent manner. Similarly, mice were successfully divided into five clusters according to diet groups (Fig. 8C), indicating that plasma amino acid profiles were altered by different protein diets. SOM showed a different classification pattern for each amino acid concentration (Supplemental Fig. S7), demonstrating the usefulness of SOM cluster analysis in governing plasma amino acid profiles.

### Association of plasma amino acid profiles with hepatic triglyceride and cholesterol contents in SOM cluster analysis

To reveal the association between plasma amino acid profiles and hepatic lipid content, a heat map of hepatic triglycerides was overlaid on the map of plasma amino acid concentrations, as shown in Fig. 8D. Members of the young P5 and middle-aged P5, P15, and P45 groups, with higher hepatic triglyceride content, clustered together (Fig. 8D). Similarly, young and middle-aged P5 mice groups with higher hepatic total cholesterol or cholesterol ester levels clustered together (Fig. 8E and G). In contrast, mice with higher hepatic free cholesterol did not cluster in the map overlaid with hepatic free cholesterol (Fig. 8F), suggesting lack of association between plasma amino acid profiles and hepatic free cholesterol. These results suggest that the plasma amino acid profiles in mice determine the hepatic contents of triglycerides, total cholesterol, and cholesterol esters.

## Discussion

The adaptive response of food intake (protein leverage) to different protein diets was elicited in both young and middle-aged mice. The low-protein diet developed mild fatty liver, with middle-aged mice showing more lipids than young mice, whereas the moderate-protein diet suppressed lipid contents and lowered the blood glucose and plasma lipid levels. Furthermore, SOM analysis of plasma amino acid concentrations in young and middle-aged mice revealed that plasma amino acid profiles varied with age and the intake of different protein diets and that liver triglyceride and cholesterol levels were associated with plasma amino acid profiles. These findings suggest that middle-aged and young mice are metabolically healthier when they consume a moderate-protein diet.

Dietary proteins provide nitrogen and amino acids for the synthesis of proteins and various nitrogen-and amino acid-related compounds in the body. A sustained supply of amino acids is required to maintain tissue and physiological functions. Therefore, the levels of amino acids in the body are tightly regulated through different nutrient sensing and signaling processes that determine protein and energy metabolism and feeding behavior [26]. In rodents, a high-protein diet (> 25 kcal%) suppresses appetite and food intake compared to a normal protein diet (10–20 kcal%). Although lean body mass is unaffected, it reduces energy intake, body weight gain, and body fat mass [27–30]. In contrast, consumption of low-protein (3–8 kcal%) or amino acid-deficient diets activates appetite-promoting pathways to compensate for protein or amino acid deficiencies by encouraging protein intake [31–34]. However, protein-deficient diets (2–3 kcal%) suppress food intake owing to anorexia, inducing decreases in body weight, body fat, lean body mass, and plasma protein concentrations [32, 35, 36]. In the present study, although aged mice consumed more food than young mice, protein leverage of food intake by different dietary protein content was elicited in both young and middle-aged animals. However, protein, fat, and carbohydrate intake were positively and negatively dependent on dietary protein content, respectively (Fig. 3). Markedly, the P5 groups did not show anorexia in either young or middle-age groups, suggesting low and inadequate protein intake, but not severe deficiency, compared with the body's demand.

Malnutrition-induced sarcopenia and frailty in elderly population are emerging problems [24]. In this study, middle-aged mice did not show reduced skeletal muscle mass compared to young mice before and after 8 weeks of feeding (Fig. 1 and 4). Although the body weights (Fig. 2C) and plasma total protein concentration (Supplemental Fig. 4E) were significantly lower in the P5 groups, there was no significant decrease in skeletal muscle mass (Fig. 4). Skeletal muscle mass is determined by the balance between synthesis and breakdown of muscle proteins. The concentration of plasma 3-methylhistidine, a marker of myolysis and a candidate marker of sarcopenia and frailty in humans [37], was measured and found significantly higher in the P5 group in both young and middle-aged mice (Supplemental Fig. 6D). Therefore, in the P5 group, protein degradation in skeletal muscle was enhanced; however, it was balanced by protein synthesis [38]. In contrast, young mice in the P45 group and middle-aged mice in the P35 and P45 groups showed significantly lower plasma 3-methylhistidine levels (Supplemental Fig. 6D), suggesting that high protein intake suppressed muscle degradation.

At the starting states of mice at 6- and 16- month-old that were reared up until then on CRF-1, the hepatic triglyceride content was significantly higher in middle-aged mice than that of young mice (Fig. 1V). At the end of the experiment, the P5 group had higher levels of triglycerides and total cholesterol in the livers of young and middle-aged mice. Overall, middle-aged mice had increased liver triglyceride levels and were more susceptible to low protein-induced steatosis than young mice (Fig. 5). Furthermore, it is noted that liver triglyceride level in middle-aged mice was decreased in P35 group compared to that at the starting state, but not in young mice. These results suggest the age-related difference in response to different diets by young and middle-aged mice. In previous studies, rats fed a low-protein diet (5% w/w) exhibited fatty liver due to increased triglyceride and total cholesterol levels in the liver [21, 25, 39]. Fatty liver formation induced by low-protein diets in growing rats is independent of changes in insulin signaling, and is mainly caused by enhanced lipid biosynthesis in the liver [39, 40]. Wali et al. reported that mice fed a low-protein diet (5 kcal%) for 18–19 weeks from the 8^th^ week of age showed higher hepatic triglyceride content than mice fed a protein 10 kcal% and 15 kcal% diet. Furthermore, gene expression analysis suggested enhanced glycerol synthesis, reduced fat oxidation, and decreased export of triglycerides into circulation via apolipoprotein B [13]. The experimental conditions used in this study were different from those used in previous studies; for example, we used mice that were not in the growth stage (6 and 16 months of age), different diet compositions (especially high lipid content) were used, and the period of breeding (8 weeks) was also different. Therefore, further analysis of the mechanism of fatty liver formation induced by a low-protein diet and age-related changes is warranted.

Solon-Biet et al*.* studied the degree of fatty liver in 15 month-old mice continuously fed diets with different percentages and amounts of protein, carbohydrate, and fat, starting from the growth phase (3 weeks) [16]. The probability of not developing a fatty liver increased when the protein intake exceeded 10 kJ/day (= 0.59 g/day, conversion factor 17 kJ/day), and decreased when the carbohydrate intake exceeded 25 kJ/day (= 1.47 g/day, conversion factor 17 kJ/g) [16, 41]. When these results were compared with the present study, the likelihood of not having fatty liver increased in the young and middle-aged mice with protein intakes of 25% (P25 group; 0.78 ± 0.04 g/day in young mice, 0.81 ± 0.02 g/day in middle-aged subjects) and higher and decreased with carbohydrate intakes of 50% (P25 group) (1.55 ± 0.07 g/day in young mice, 1.62 ± 0.05 g/day in middle-aged mice) and lower. In other words, the P5 group was most likely to develop fatty liver, which is consistent with the mild fat deposition in this study. In addition, the P5 group of middle-aged mice had a higher intake of fat and carbohydrates than the younger group, which may partly explain higher triglyceride levels in the liver. In contrast, under the present experimental conditions, triglyceride levels in the liver of the P45 group was slightly higher in both young and middle-aged animals, indicating that the moderate-protein intake group, with the lowest level of liver triglycerides, was metabolically healthy (Fig. 5).

The concentration of amino acids in blood is determined by the balance between influx (amino acid intake, NEAA synthesis, elimination from cells, and muscle protein degradation) and efflux (urinary excretion, degradation, cellular uptake, and muscle protein synthesis). Amino acid uptake and efflux from cells is carried out by a variety of solute carrier proteins that transport different amino acids [42]. Therefore, the concentrations of individual amino acids in the plasma did not correspond to the amino acid content of the feed. In the present study, plasma amino acid concentrations differed significantly for each amino acid in the groups (Fig. 7 and Supplemental Fig. 7), making it difficult to interpret the effects of diets with different protein percentages and ages on plasma amino acid concentrations. Plasma BCAA levels were higher in the P35 group in young and middle-aged mice (Fig. 7X). Since BCAA is a component of muscle proteins and acts as a signal for protein synthesis, the P35 group was in a state of high reserve strength. Although plasma EAA concentrations were maintained in all the groups, plasma NEAA concentrations were largely affected by dietary differences. Plasma NEAA concentrations were higher in the P5 group in both young and middle-aged mice, suggesting enhancement of NEAA synthesis to compensate for the lack of amino acid intake owing to the low-protein diet. However, plasma NEAA levels were lower in the P45 group in young and middle-aged mice. On one hand, as dietary protein content increased, the young and middle-aged mice showed increased protein intake but decreased carbohydrate intake (Fig. 3E, F, K and L), leading to degradation of excessive amino acids, subsequent gluconeogenesis, and glucose production in the liver to meet the whole-body energy demand (especially in P45 groups). Thus, it is less necessary for mice to transition toward the use of lipid oxidation as a fuel. This interpretation is supported by unchanged levels of total ketone bodies (Fig. 6E), triglycerides (Fig. 6B), and increasing urea over the P15 diet in the plasma (Fig. 6F), that is, increased gluconeogenesis due to higher mitochondrial amino acid degradation in the liver activates the urea cycle to regulate pH by excessive ammonification. On the other hand, as dietary protein content decreased, the young and middle-aged mice showed decreased protein intake but increased carbohydrate intake (Fig. 3E, F, K and L), leading to the suppression of amino acid degradation, glycolysis, and subsequent NEAA production (Fig. 7W) in the liver to meet amino acid demand for the whole-body (especially in P5 groups). This interpretation is supported by the lack of higher blood glucose levels (Fig. 6A) and decreased plasma urea levels in the P5 diet (Fig. 6F). These results suggest that the moderate-protein diet is metabolically well-balanced for plasma biochemical parameters in young and middle-aged mice.

Liver triglyceride levels can be predicted from the serum amino acid profile using SOM and multi-layer perceptron analyses [21]. They integrated the serum amino acid concentrations of each of the 20 amino acids in rats fed normal (15% w/w), low-protein (5% w/w), or amino acid-deficient diets for 1 week from 5^th^ week of age. In the present study, SOM analysis revealed the formation of multiple clusters consisting of individual mice with similar plasma amino acid profiles that were clearly classified according to age and diet (Fig. 8). This finding indicates that the plasma amino acid concentration profile is determined by the age of the animals and the percentage of protein in their diet. The clusters were also classified according to liver triglyceride content (Fig. 8D), indicating that liver triglyceride content corresponded to plasma amino acid concentration profiles in young and middle-aged mice fed diets with different protein percentages. However, it is unclear how aging and diets with different protein percentages regulate plasma amino acid concentration profiles and liver lipid content, and thus requires further analysis.

Recent reports have shown that lifelong protein restriction extends lifespan and promotes metabolic health [11–13, 15, 16, 19]. However, note that extending the lifespan and maintaining metabolic health (or extending the healthspan) are different life phenomena, and age-specific nutritional (particularly proteins) requirements to maintain metabolic health may vary with life stages. Our finding that aged mice performed better on a higher protein diet is consistent with the analysis of age-specific mortality across the life course by Senior et al*.* [12] for mice in the experiment first described by Solon-Biet et al*.* [16]. These studies showed that lifespan was longest with lower protein and higher carbohydrate diets when mice were confined throughout life, but that age-specific mortality risk changes as a function of macronutrient composition. Mice died at a greater rate on a higher-protein diet during midlife, but survivors did better beyond age 16–20 months of age on a higher-protein diet. Furthermore, analysis of the same mice showed that reproductive potential was higher in higher protein diets [14]. Hence, protein requirements change through the course of life, being higher in younger reproductive mice, reducing through middle age, and rising again in older mice as protein efficiency declines. The same pattern is likely to be observed in humans [15]. It is difficult to compare the outcomes of our study with those of previous studies because of various differences in the experimental design and focused parameters.

Our results indicate that moderate-protein intake, at least beyond a certain amount, is appropriate to maintain metabolic health (liver and plasma lipid levels and blood glucose levels) during approach to old age, similar to that in young mice. Furthermore, plasma amino acid profiles, which varied with age and protein intake, were found to be associated with liver lipid content using SOM analysis, which would be an important interventional target for the control of liver triglyceride and cholesterol levels.

The limitations of the present study are as follows, (i) we only used young male and middle-aged C57BL/6NCr mice. Therefore, we cannot discuss differences in sex and genetic background as reported by Green et al. [19]. (ii) The results in the present study are relevant to mice that have been reared until 6 and 16 months on CRF-1, thus might differ in mice reared up to 6 and 16 months on different diet formulations. (iii) Skeletal muscle mass did not decrease in middle-aged mice in the P5 group. To investigate the relationship between malnutrition-induced sarcopenia and frailty in the future, muscle strength, physical function, and activity should be measured in animals fed diets with lower protein percentages for more than 8 weeks. (iv) Casein, an animal protein, was used as the feed protein. However, the results may differ depending on the nature and source of protein. Diets with 25% lipids were used, considering the percentage of dietary lipid intake in humans; however, the results may differ if a lower lipid percentage is used. (v) Pathological analyses of the kidneys were performed to assess the risk of kidney burden due to high protein intake. However, other indicators of kidney function should also be examined and considered. (vi) In the present study, we did not measure the adipose tissue weight; therefore, the systemic distribution of lipids remains unknown.

### Supplementary information

Below is the link to the electronic supplementary material.

Supplementary file1 (PDF 5530 KB)

## Data Availability

The data that support the findings of this study are available from the corresponding author upon reasonable request.

## References

[1] Blagosklonny MV (2021). The goal of geroscience is life extension. Oncotarget.

[2] Kaeberlein M (2018). How healthy is the healthspan concept?. GeroScience.

[3] Kumar S, Peterson TR (2020). Moonshots for aging. Nutr Healthy. Aging.

[4] Marzetti E, Calvani R, Tosato M, Cesari M, Di Bari M, Cherubini A, Collamati A, D’Angelo E, Pahor M, Bernabei R, Landi F, Consortium S. Sarcopenia: an overview. Aging Clin Exp Res. 2017;29:11-7. 10.1007/s40520-016-0704-5 28155183

[5] Fried LP, Ferrucci L, Darer J, Williamson JD, Anderson G (2004). Untangling the concepts of disability, frailty, and comorbidity: implications for improved targeting and care. J Gerontol A Biol Sci Med Sci.

[6] Fried LP, Tangen CM, Walston J, Newman AB, Hirsch C, Gottdiener J, Seeman T, Tracy R, Kop WJ, Burke G, McBurnie MA, Cardiovascular Health Study Collaborative Research G (2001). Frailty in older adults: evidence for a phenotype. J Gerontol A Biol Sci Med Sci.

[7] Hamerman D (1999). Toward an understanding of frailty. Ann Intern Med.

[8] Rowe IA, Wong VW, Loomba R (2022). Treatment Candidacy for Pharmacologic Therapies for NASH. Clin Gastroenterol Hepatol.

[9] Weindruch R, Walford RL, Fligiel S, Guthrie D (1986). The retardation of aging in mice by dietary restriction: longevity, cancer, immunity and lifetime energy intake. J Nutr.

[10] Cummings NE, Lamming DW (2017). Regulation of metabolic health and aging by nutrient-sensitive signaling pathways. Mol Cell Endocrinol.

[11] Richardson NE, Konon EN, Schuster HS, Mitchell AT, Boyle C, Rodgers AC, Finke M, Haider LR, Yu D, Flores V, Pak HH, Ahmad S, Ahmed S, Radcliff A, Wu J, Williams EM, Abdi L, Sherman DS, Hacker T, Lamming DW (2021). Lifelong restriction of dietary branched-chain amino acids has sex-specific benefits for frailty and lifespan in mice. Nat Aging.

[12] Senior AM, Solon-Biet SM, Cogger VC, Le Couteur DG, Nakagawa S, Raubenheimer D, Simpson SJ (2019). Dietary macronutrient content, age-specific mortality and lifespan. Proc Biol Sci.

[13] Wali JA, Milner AJ, Luk AWS, Pulpitel TJ, Dodgson T, Facey HJW, Wahl D, Kebede MA, Senior AM, Sullivan MA, Brandon AE, Yau B, Lockwood GP, Koay YC, Ribeiro R, Solon-Biet SM, Bell-Anderson KS, O'Sullivan JF, Macia L, Forbes JM, Cooney GJ, Cogger VC, Holmes A, Raubenheimer D, Le Couteur DG, Simpson SJ (2021). Impact of dietary carbohydrate type and protein-carbohydrate interaction on metabolic health. Nat Metab.

[14] Solon-Biet SM, Walters KA, Simanainen UK, McMahon AC, Ruohonen K, Ballard JW, Raubenheimer D, Handelsman DJ, Le Couteur DG, Simpson SJ (2015). Macronutrient balance, reproductive function, and lifespan in aging mice. Proc Natl Acad Sci U S A.

[15] Levine ME, Suarez JA, Brandhorst S, Balasubramanian P, Cheng CW, Madia F, Fontana L, Mirisola MG, Guevara-Aguirre J, Wan J, Passarino G, Kennedy BK, Wei M, Cohen P, Crimmins EM, Longo VD (2014). Low protein intake is associated with a major reduction in IGF-1, cancer, and overall mortality in the 65 and younger but not older population. Cell Metab.

[16] Solon-Biet SM, McMahon AC, Ballard JW, Ruohonen K, Wu LE, Cogger VC, Warren A, Huang X, Pichaud N, Melvin RG, Gokarn R, Khalil M, Turner N, Cooney GJ, Sinclair DA, Raubenheimer D, Le Couteur DG, Simpson SJ (2014). The ratio of macronutrients, not caloric intake, dictates cardiometabolic health, aging, and longevity in ad libitum-fed mice. Cell Metab.

[17] Galbreath M, Campbell B, LaBounty P, Bunn J, Dove J, Harvey T, Hudson G, Gutierrez JL, Levers K, Galvan E, Jagim A, Greenwood L, Cooke MB, Greenwood M, Rasmussen C, Kreider RB. Effects of adherence to a higher protein diet on weight loss, markers of health, and functional capacity in older women participating in a resistance-based exercise program. nutrients. 2018;10:1070. 10.3390/nu10081070 PMC6115985 30103509

[18] Verspoor E, Voortman T, van Rooij FJA, Rivadeneira F, Franco OH, Kiefte-de Jong JC, Schoufour JD (2020). Macronutrient intake and frailty: the Rotterdam Study. Eur J Nutr.

[19] Green CL, Pak HH, Richardson NE, Flores V, Yu D, Tomasiewicz JL, Dumas SN, Kredell K, Fan JW, Kirsh C, Chaiyakul K, Murphy ME, Babygirija R, Barrett-Wilt GA, Rabinowitz J, Ong IM, Jang C, Simcox J, Lamming DW (2022). Sex and genetic background define the metabolic, physiologic, and molecular response to protein restriction. Cell Metab.

[20] Hahn O, Drews LF, Nguyen A, Tatsuta T, Gkioni L, Hendrich O, Zhang Q, Langer T, Pletcher S, Wakelam MJO, Beyer A, Gronke S, Partridge L (2019). A nutritional memory effect counteracts benefits of dietary restriction in old mice. Nat Metab.

[21] Nishi H, Yamanaka D, Kamei H, Goda Y, Kumano M, Toyoshima Y, Takenaka A, Masuda M, Nakabayashi Y, Shioya R, Kataoka N, Hakuno F, Takahashi SI (2018). Importance of Serum Amino Acid Profile for Induction of Hepatic Steatosis under Protein Malnutrition. Sci Rep.

[22] Folch J, Lees M, Sloane Stanley GH (1957). A simple method for the isolation and purification of total lipides from animal tissues. J Biol Chem.

[23] Shimbo K, Kubo S, Harada Y, Oonuki T, Yokokura T, Yoshida H, Amao M, Nakamura M, Kageyama N, Yamazaki J, Ozawa S, Hirayama K, Ando T, Miura J, Miyano H (2010). Automated precolumn derivatization system for analyzing physiological amino acids by liquid chromatography/mass spectrometry. Biomed Chromatogr.

[24] Verlaan S, Ligthart-Melis GC, Wijers SLJ, Cederholm T, Maier AB, de van der Schueren MAE. High prevalence of physical frailty among community-dwelling malnourished older adults-a systematic review and meta-analysis. J Am Med Dir Assoc. 2017;18:374–82. 10.1016/j.jamda.2016.12.074 28238676

[25] Toyoshima Y, Tokita R, Taguchi Y, Akiyama-Akanishi N, Takenaka A, Kato H, Chida K, Hakuno F, Minami S, Takahashi S (2014). Tissue-specific effects of protein malnutrition on insulin signaling pathway and lipid accumulation in growing rats. Endocr J.

[26] Tome D, Chaumontet C, Even PC, Darcel N, Thornton SN, Azzout-Marniche D (2020). Protein Status Modulates an Appetite for Protein To Maintain a Balanced Nutritional State-A Perspective View. J Agric Food Chem.

[27] Bensaid A, Tome D, L'Heureux-Bourdon D, Even P, Gietzen D, Morens C, Gaudichon C, Larue-Achagiotis C, Fromentin G (2003). A high-protein diet enhances satiety without conditioned taste aversion in the rat. Physiol Behav.

[28] Chaumontet C, Even PC, Schwarz J, Simonin-Foucault A, Piedcoq J, Fromentin G, Azzout-Marniche D, Tome D (2015). High dietary protein decreases fat deposition induced by high-fat and high-sucrose diet in rats. Br J Nutr.

[29] Jean C, Rome S, Mathe V, Huneau JF, Aattouri N, Fromentin G, Achagiotis CL, Tome D (2001). Metabolic evidence for adaptation to a high protein diet in rats. J Nutr.

[30] Vu JP, Luong L, Parsons WF, Oh S, Sanford D, Gabalski A, Lighton JR, Pisegna JR, Germano PM (2017). Long-Term Intake of a High-Protein Diet Affects Body Phenotype, Metabolism, and Plasma Hormones in Mice. J Nutr.

[31] Carreiro AL, Dhillon J, Gordon S, Higgins KA, Jacobs AG, McArthur BM, Redan BW, Rivera RL, Schmidt LR, Mattes RD (2016). The Macronutrients, Appetite, and Energy Intake. Annu Rev Nutr.

[32] Du F, Higginbotham DA, White BD (2000). Food intake, energy balance and serum leptin concentrations in rats fed low-protein diets. J Nutr.

[33] Pezeshki A, Chelikani PK (2021). Low Protein Diets and Energy Balance: Mechanisms of Action on Energy Intake and Expenditure. Front Nutr.

[34] Sorensen A, Mayntz D, Raubenheimer D, Simpson SJ (2008). Protein-leverage in mice: the geometry of macronutrient balancing and consequences for fat deposition. Obesity (Silver Spring).

[35] Chaumontet C, Recio I, Fromentin G, Benoit S, Piedcoq J, Darcel N, Tome D (2018). The Protein Status of Rats Affects the Rewarding Value of Meals Due to their Protein Content. J Nutr.

[36] Moro J, Chaumontet C, Even PC, Blais A, Piedcoq J, Gaudichon C, Tome D, Azzout-Marniche D (2021). Severe protein deficiency induces hepatic expression and systemic level of FGF21 but inhibits its hypothalamic expression in growing rats. Sci Rep.

[37] Kochlik B, Stuetz W, Peres K, Feart C, Tegner J, Rodriguez-Manas L, Grune T, Weber D. Associations of plasma 3-methylhistidine with frailty status in french cohorts of the FRAILOMIC initiative. J Clin Med. 2019;8:1010. 10.3390/jcm8071010 PMC6678434 31295923

[38] Mittendorfer B, Klein S, Fontana L (2020). A word of caution against excessive protein intake. Nat Rev Endocrinol.

[39] Otani L, Nishi H, Koyama A, Akasaka Y, Taguchi Y, Toyoshima Y, Yamanaka D, Hakuno F, Jia H, Takahashi SI, Kato H (2020). Low-arginine and low-protein diets induce hepatic lipid accumulation through different mechanisms in growing rats. Nutr Metab (Lond).

[40] Taguchi Y, Toyoshima Y, Tokita R, Kato H, Takahashi SI, Minami S (2017). Triglyceride synthesis in hepatocytes isolated from rats fed a low-protein diet is enhanced independently of upregulation of insulin signaling. Biochem Biophys Res Commun.

[41] Simpson SJ, Raubenheimer D, Cogger VC, Macia L, Solon-Biet SM, Le Couteur DG, George J (2018). The nutritional geometry of liver disease including non-alcoholic fatty liver disease. J Hepatol.

[42] Kandasamy P, Gyimesi G, Kanai Y, Hediger MA (2018). Amino acid transporters revisited: New views in health and disease. Trends Biochem Sci.

