# Supplementary material for: Moderate protein intake percentage in mice for maintaining metabolic health during approach to old age
Source: GeroScience. 2023 Apr 28;45(4):2707–26. doi: 10.1007/s11357-023-00797-3 (PMC10651611; doi:10.1007/s11357-023-00797-3)
Supplement: Supplementary file 1 — Supplementary file1 (PDF 5530 KB) [file 11357_2023_797_MOESM1_ESM.pdf]

Supplementary Information

Moderate protein intake percentage in mice for maintaining metabolic health during approach to old age

GeroScience

Yoshitaka Kondo[1, 2], Hitoshi Aoki[3], Masato Masuda[4], Hiroki Nishi[4], Yoshihiro Noda[5], Fumihiko Hakuno[4], Shin-Ichiro Takahashi[4], Takuya Chiba[2], and Akihito Ishigami[1, *]

[1]Molecular Regulation of Aging, Tokyo Metropolitan Institute of Gerontology, Tokyo 173-0015, Japan.
[2]Biomedical Gerontology Laboratory, Faculty of Human Sciences, Waseda University, Saitama 359-1192, Japan.
[3]Research and Development Division, Nichirei Foods Inc., Chiba 261-0002, Japan.
[4]Department of Animal Sciences and Applied Biological Chemistry, Graduate School of Agricultural and Life Sciences, University of Tokyo, Tokyo, Japan.
[5]Department of Animal Facility, Tokyo Metropolitan Institute of Gerontology, Tokyo 173-0015, Japan.

***Corresponding author:** Akihito Ishigami, Ph.D., Molecular Regulation of Aging, Tokyo Metropolitan Institute of Gerontology (TMIG), 35-2 Sakae-cho, Itabashi-ku, Tokyo 173-0015, Japan. Phone +81-3-3964-3241, E-mail: ishigami@tmig.or.jp

Supplemental Fig. 1-7

Supplemental Table 1

Supplemental Fig. 1

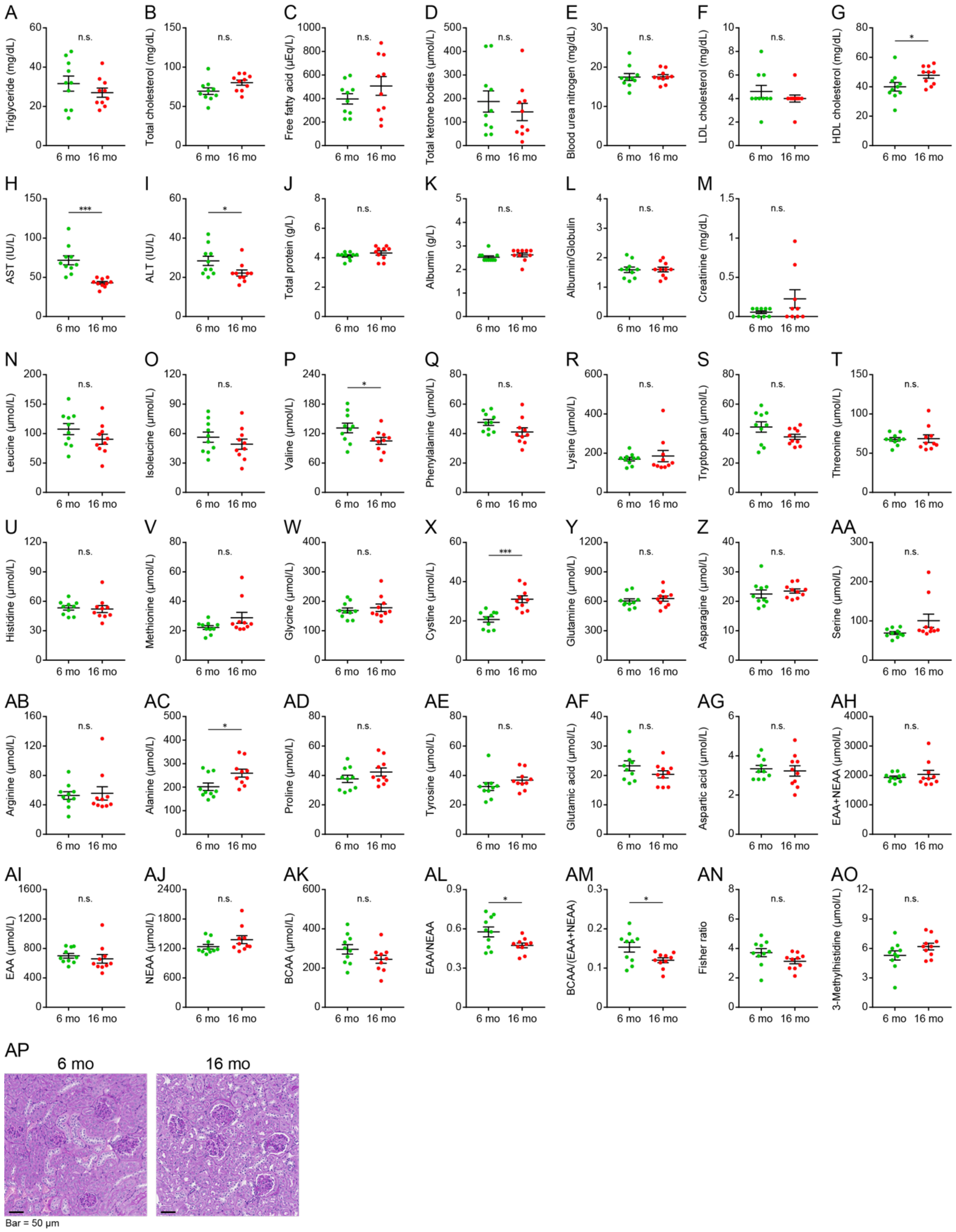

Supplemental Fig. 1. Blood biochemical parameters and plasma amino acid concentrations in young and middle-aged mice. Plasma concentrations of (A) triglycerides, (B) total cholesterol, (C) free fatty acids, (D) total ketone bodies, and (E) blood urea nitrogen in young and middle-aged mice (n = 10 per group). Plasma levels of (F) low-density lipoprotein (LDL) cholesterol, (G) high-density lipoprotein (HDL) cholesterol, (H) aspartate aminotransferase (AST), (I) alanine aminotransferase (ALT), (J) total

protein, (K) albumin, (L) albumin/globulin, and (M) creatinine in young and middle-aged mice (n = 10 per group). Plasma concentrations of (N) leucine, (O) isoleucine, (P) valine, (Q) phenylalanine, (R) lysine, (S) tryptophan, (T) threonine, (U) histidine, (V) methionine, (W) glycine, (X) cystine, (Y) glutamine, (Z) asparagine, (AA) serine, (AB) arginine, (AC) alanine, (AD) proline, (AE) tyrosine, (AF) glutamic acid, and (AG) aspartic acid. Plasma concentrations of (AH) essential amino acids (EAAs) and non-essential amino acids (NEAAs), (AI) EAA, (AJ) NEAA, and (AK) branched-chain amino acids (BCAA) in young and middle-aged mice (n = 10 per group). (AL) Ratio of EAAs to NEAAs. (AM) Ratio of BCAAs to essential- and non-essential amino acids (EAA+NEAA). (AN) Fisher ratio (ratio of BCAAs to aromatic amino acids) and (AO) 3-methylhistidine concentration in young and middle-aged mice (n = 10 per group). (AP) Representative images of periodic acid-Schiff (PAS) staining of kidney sections from young and middle-aged mice. Data are presented as mean ± SEM and analyzed using the Welch's t-test (A-AO). n.s., not significant. $^{*}p < 0.05$, $^{***}p < 0.001$

Supplemental Fig. 2

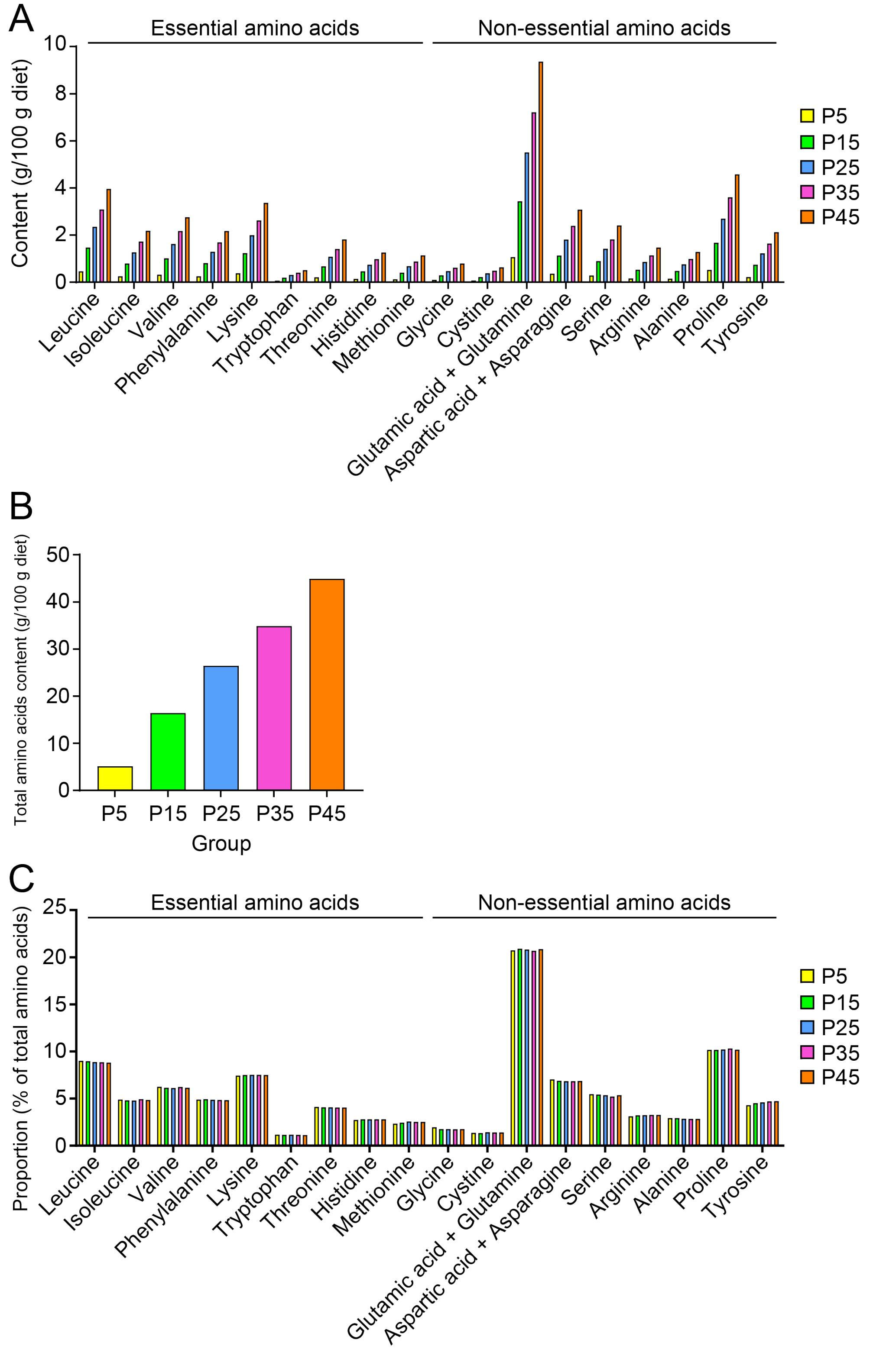

Supplemental Fig. 2 Amino acid contents of diets with different protein percentages. (A) Amino acid contents of diets (n = 1) with different protein percentages P5, P15, P25, P35, and P45 groups were fed with 5, 15, 25, 35, or 45 kcal% protein, 70, 60, 50, 40, or 30 kcal% carbohydrate, respectively, and 25 kcal% fat. (B) The total amino acid content in groups fed with different diets. (C) The proportions of amino acids in total amino acids content in different diet groups were similar to those of casein protein

Supplemental Fig. 3

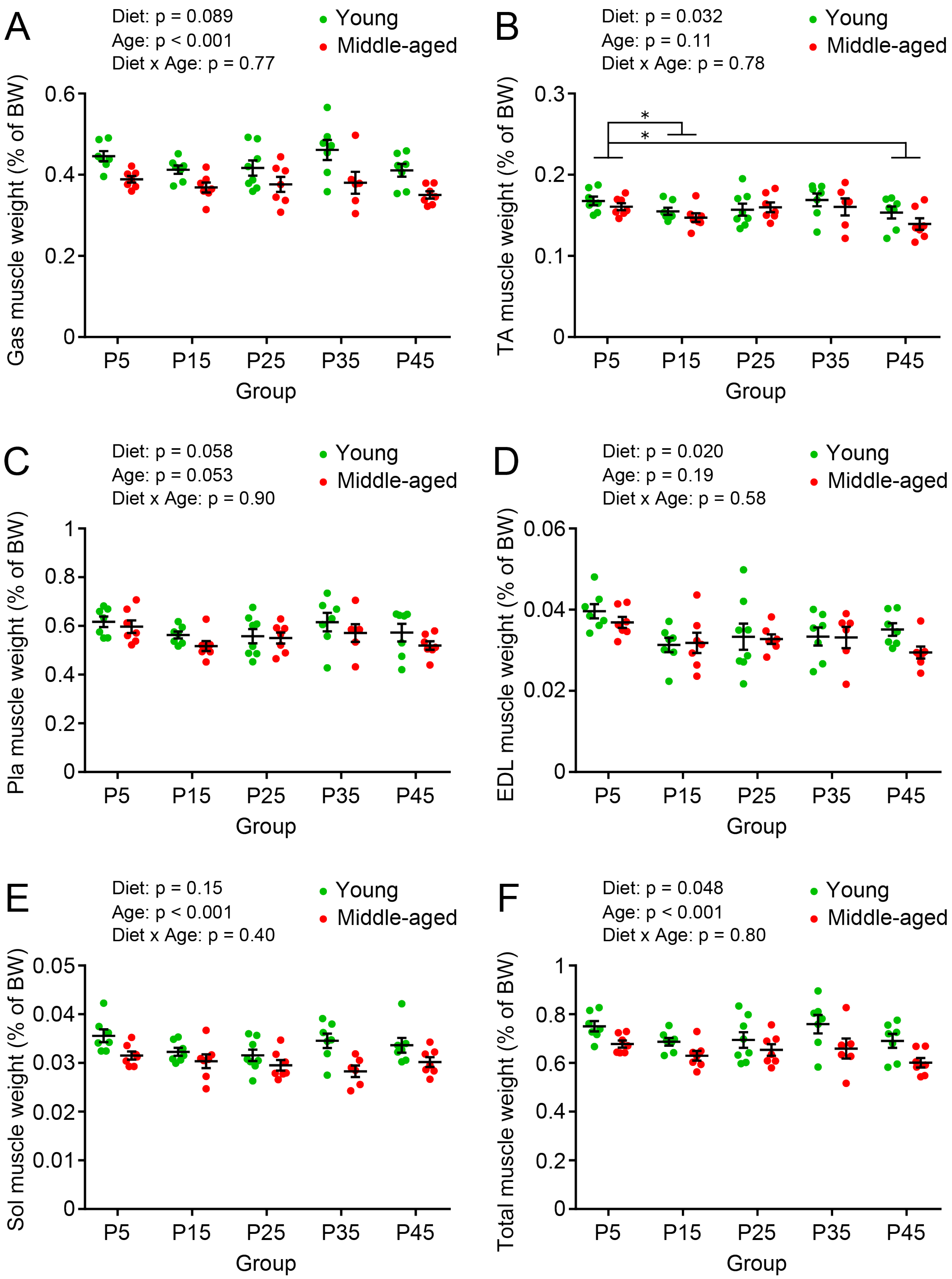

Supplemental Fig. 3 Skeletal muscle weights normalized by bodyweight of young and middle-aged mice fed with different protein diets. Weights normalized by body weight of (A) gastrocnemius (Gas) muscle, (B) tibialis anterior (TA) muscle, (C) plantaris (Pla) muscle, (D) extensor digitorum longus (EDL) muscle, (E) soleus (Sol) muscle, and (F) total weight of the five muscles of young and middle-aged mice (n = 6-8 per group) after 8 weeks of feeding. Data are presented as mean ± SEM and analyzed using two-way ANOVA followed by Tukey post-test to examine the effects of diet (A-F). $^{*}p < 0.05$

Supplemental Fig. 4

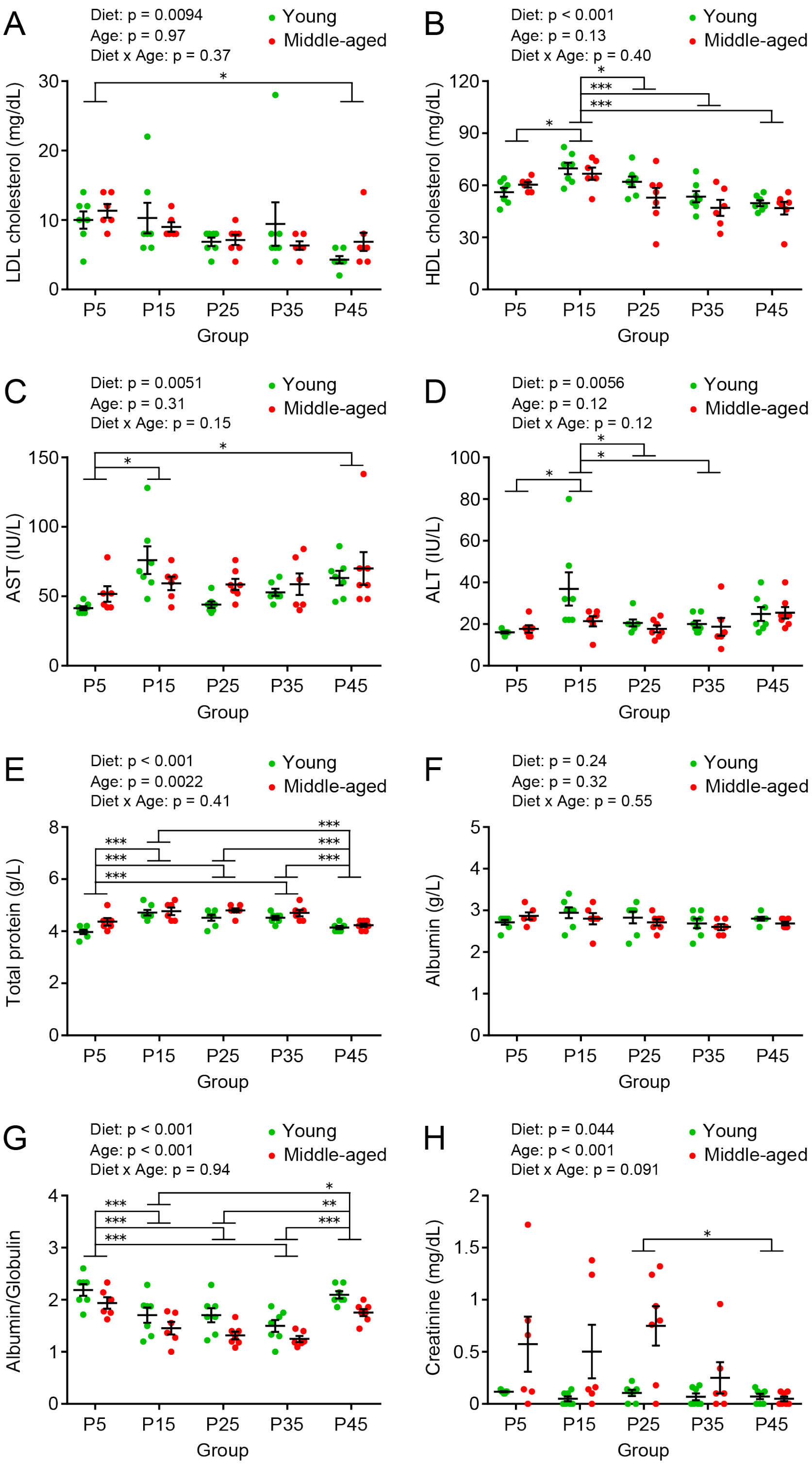

Supplemental Fig. 4. Blood biochemical parameters in young and middle-aged mice fed with different protein diets. Plasma levels of (A) low-density lipoprotein (LDL) cholesterol, (B) high-density lipoprotein (HDL) cholesterol, (C) aspartate aminotransferase (AST), (D) alanine aminotransferase (ALT), (E) total protein, (F) albumin, (G) albumin/globulin, and (H) creatinine in young and middle-aged mice (n = 6-7 per group) after eight weeks of feeding. Data are presented as mean ± SEM and analyzed using two-way ANOVA followed by Tukey post-test to examine the effects of diet (A-H). $^{*}p < 0.05$, $^{**}p < 0.01$, $^{***}p < 0.001$

Supplemental Fig. 5

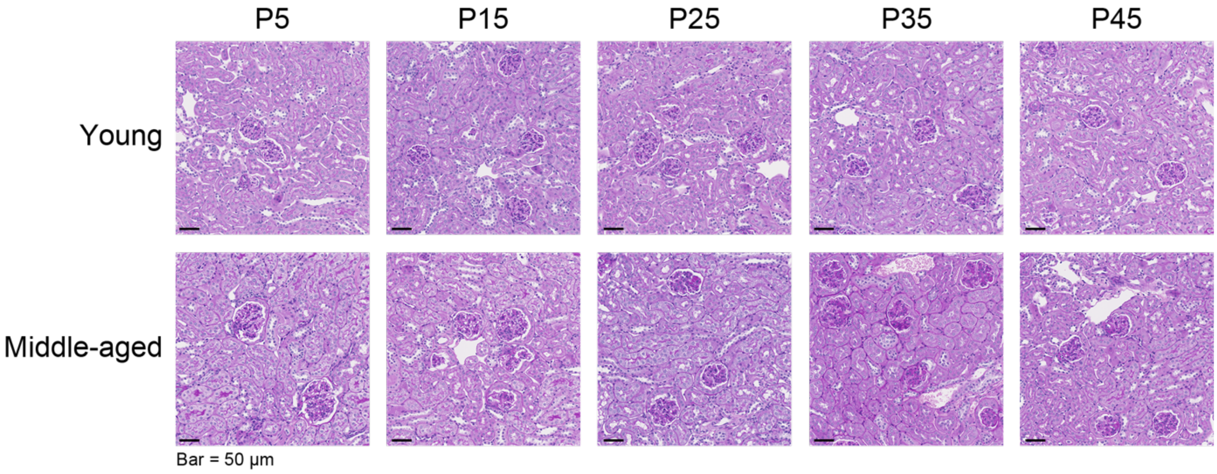

Supplemental Fig. 5 Histology of kidney sections in young and middle-aged mice fed with different protein diets. Representative images of periodic acid-Schiff (PAS) staining of kidney sections from young and middle-aged mice (n = 6-8 per group) fed diets with different protein content after eight weeks of feeding

Supplemental Fig. 6

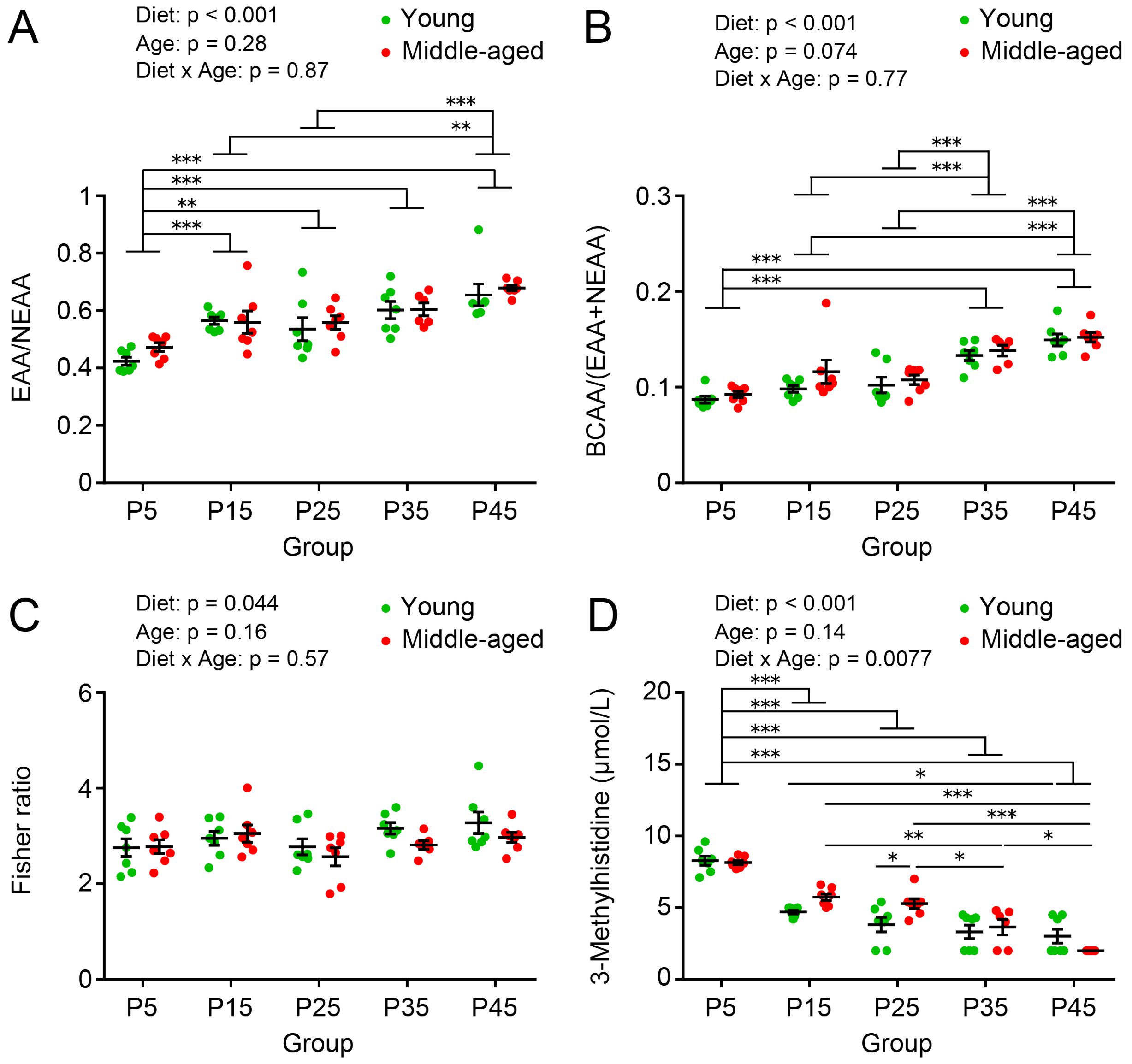

Supplemental Fig. 6 Characteristic of plasma amino acids in young and middle-aged mice fed with different protein diets. (A) The ratio of the essential amino acids (EAAs) to non-essential amino acids (NEAAs). (D) The ratio of the branched-chain amino acids (BCAAs) to essential- and non-essential amino acid (EAA+NEAA). (C) Fisher ratio (the ratio of the BCAAs to aromatic amino acids) and (D) 3-methylhistidine concentration in the young and middle-aged mice (n = 6-7 per group) after 8 weeks of feeding. Data are presented as mean ± SEM and analyzed using two-way ANOVA followed by a Sidak post-test to examine the effect of age or Tukey post-test to examine the effects of diet (A-D). $^{*}p < 0.05$, $^{**}p < 0.01$, $^{***}p < 0.001$

Supplemental Fig. 7

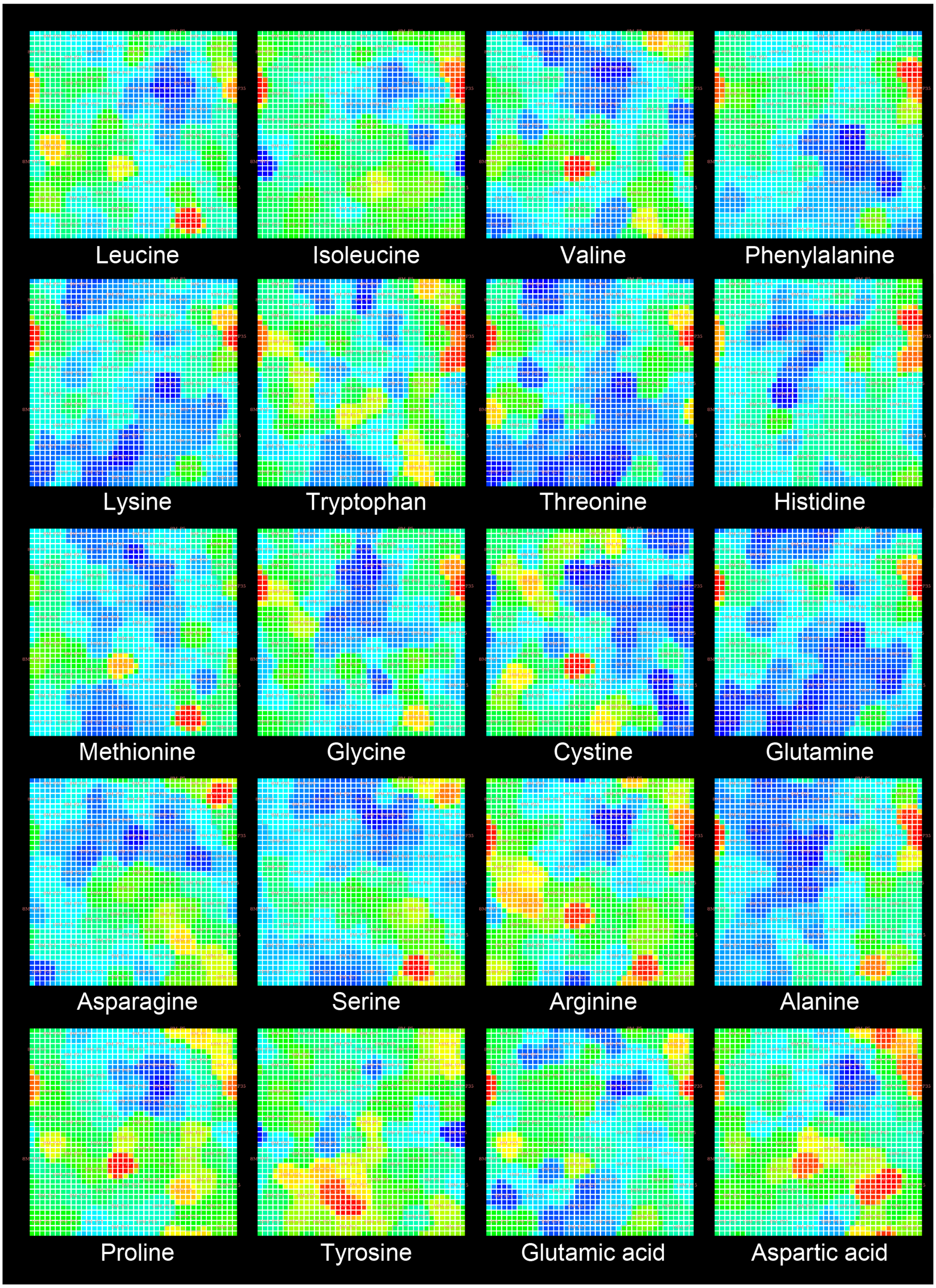

Supplemental Fig. 7 Self-organizing map (SOM) of plasma amino acid profiles overlaid with each amino acid concentration in young and middle-aged mice fed with different protein diets. Heat maps of

plasma leucine, isoleucine, valine, phenylalanine, lysine, tryptophan, threonine, histidine, methionine, glycine, cystine, glutamine, asparagine, serine, arginine, alanine, proline, tyrosine, glutamic acid, and aspartic acid concentrations are overlaid on the map shown in Fig. 5A. Colors represent the measured normalized values of plasma amino acid concentration (red, high = 1; blue, low = 0). Data sets of plasma amino acid concentrations were obtained from 69 young and middle-aged mice (n = 6-7 per group) after eight weeks of feeding

Supplemental Table 1 Diet composition of CRF-1

Group	CRF-1	
	g%	kcal%
Moisture	8.2	0
Crude protein	21.9	24.5
Crude fat	5.4	13.6
Crude ash	6.3	0
Crude fiber	2.9	0
Nitrogen-free extract	55.3	61.9
Total	100.0	100.0
kcal/g	3.6	3.6

Vitamins	in 100 g	
Vitamin A	3245	IU
Vitamin D3	643	IU
Vitamin E	20.3	mg
Vitamin K3	0.16	mg
Vitamin B1	4.74	mg
Vitamin B2	3.31	mg
Vitamin C	12	mg
Vitamin B6	1.27	mg
Vitamin B12	17.2	μg
Inositol	453	mg
Biotin	27.1	μg
Pantothenic acid	5.07	mg
Niacin	15.62	mg
Choline	0.26	g
Folic acid	0.27	mg

Minerals	in 100 g	
Ca	1.22	g
P	0.81	g
Mg	0.23	g
Na	0.26	g
K	0.86	g
Fe	13.8	mg
Al	2.1	mg
Cu	0.95	mg
Zn	6.15	mg
Co	0.33	mg
Mn	7.27	mg
Ca/P	1.5	
Ca/Mg	5.2	
K/Na	3.27	

Amino acid	in 100 g	
Isoleucine	0.82	g
leucine	1.61	g
lysine	1.12	g
Methionine	0.44	g
Cystine	0.34	g
Phenylalanine	0.96	g
Tyrosine	0.64	g
Threonine	0.83	g
Tryptophan	0.27	g
Valine	1.02	g
Arginine	1.3	g
Histidine	0.56	g
Alanine	1.13	g
Aspartic acid	1.92	g
Glutamic acid	3.75	g
Glycine	1.1	g
Proline	1.26	g
Serine	1.03	g
